# Spectrally Resolved Fundus Autofluorescence in Healthy Eyes: Repeatability and Topographical Analysis of the Green-Emitting Fluorophores

**DOI:** 10.3390/jcm9082388

**Published:** 2020-07-27

**Authors:** Enrico Borrelli, Marco Battista, Biancamaria Zuccaro, Riccardo Sacconi, Maria Brambati, Lea Querques, Francesco Prascina, SriniVas R. Sadda, Francesco Bandello, Giuseppe Querques

**Affiliations:** 1 Department of Ophthalmology, University Vita-Salute, IRCCS Ospedale San Raffaele, 20132 Milan, Italy; borrelli.enrico@yahoo.com (E.B.); marco.battista91@gmail.com (M.B.); biancazuccaro@hotmail.it (B.Z.); ric.sacconi@gmail.com (R.S.); brambati.maria@hsr.it (M.B.); querques.lea@hsr.it (L.Q.); francescoprascina@libero.it (F.P.); bandello.francesco@hsr.it (F.B.); 2 Doheny Image Reading Center, Doheny Eye Institute, Los Angeles, CA 90033, USA; ssadda@doheny.org; 3 Department of Ophthalmology, David Geffen School of Medicine at UCLA, Los Angeles, CA 90095, USA

**Keywords:** autofluorescence, green-emitting fluorophores, metabolic study

## Abstract

The aim of this study was to report normal measurements of green-emitting fluorophores in the macula of healthy young individuals and to assess the repeatability of these quantitative metrics. To do so, healthy young volunteers were imaged twice (7 ± 3 days apart) using a confocal blue-light fundus autofluorescence (FAF) device with a shorter excitation wavelength (peak at 450 nm) and the capability for separately detecting the red and green components of the emission spectrum. The main outcome measure was the percentage of area occupied by green-emitting fluorophores in the macula. In addition, this measure was performed in separate regions providing a topographical assessment in the foveal, parafoveal and perifoveal regions. Furthermore, the level of agreement between repeated measurements was evaluated. Thirty eyes from 30 healthy volunteers were included in this analysis. Mean age was 26.2 ± 2.8 years (median: 25.0 years; range: 23.0–32.0 years). Median (interquartile range—IQR) area occupied by green-emitting fluorophores was 3.6% (1.9–4.7%) in the macular region. In the topographical analysis, this percentage was higher in the foveal area (median = 33.3%, IQR = 21.9–41.2%), as compared with both the parafoveal (median = 5.3%; IQR = 2.4–8.1%; *p* < 0.0001) and perifoveal (median = 0.5%, IQR = 0.2–0.8%; *p* < 0.0001) regions. The coefficient of variation (CV; ranging from 1.1% to 1.7% in the analyzed regions) and the intraclass correlation coefficient (ICC; ranging from 0.93 to 0.97) indicated high levels of repeatability. In conclusion, the assessment of green-emitting fluorophores is repeatable. The distribution of these fluorophores is highest in the foveal region. Assuming that flavin adenine dinucleotide (FAD) emits in the green autofluorescence spectrum, this variability could be secondary to an increased quantity of mitochondria in the foveal region.

## 1. Introduction

Fundus autofluorescence (FAF) is a non-invasive imaging modality which is used to record the retinal and retinal pigment epithelial (RPE) autofluorescence [1,2,3]. Lipofuscin, which is a mixture of fluorophores, is considered to be the most intense source of autofluorescence in the posterior eye [1,2]. Consequently, short wavelength FAF is mainly thought to be an effective surrogate for lipofuscin concentration in the RPE. Importantly, lipofuscin is known to emit in the long-wavelength emission range between 560 and 700 nm (red spectrum), and thus represents a red-emitting fluorophore [4]. 

When the retina is excited with a blue light, other minor fluorophores in addition to lipofuscin contribute to the detected fluorescence of the posterior eye. Importantly, these minor fluorophores emit in the short-wavelength emission range (510–560 nm, green spectrum) [4]. These green-emitting fluorophores include coenzymes in redox reactions such as flavin adenine dinucleotide (FAD), advanced glycation end products (AGE), and collagen/elastin [5]. Notably, the excitation and emission spectra of these green-emitting fluorophores have been extensively characterized.^4^ Importantly, they are known to emit a very weak fluorescence intensity and a strategy to increase the activation of these green-emitting fluorophores is to employ a lower wavelength (~450 nm) to excite the retina [4,6,7,8]. 

The combination of a lower excitation blue-light wavelength that further excites green-emitting fluorophores with the ability to isolate the green and red-emitting spectral components (or green- and red-emission fluorescence components, GEFC and REFC, respectively) has recently allowed green-emitting fluorophores to be studied. Using a new spectrally resolved (or “color-coded”) confocal FAF device (Eidon, CenterVue, Padua, Italy), previous studies have characterized the presence of the green-emitting fluorophores in diseased eyes [7,8,9,10]. However, detailed description of the distribution of these green-emitting fluorophores in healthy eyes has not been performed. First establishing spectrally resolved FAF reference parameters in the healthy macula, however, is essential in order to better understand the alterations caused by disease. In addition, the repeatability of these measurements must be established in order to define the magnitude of change which represents a significant alteration.

The aim of this study was thus to report normal measurements of green-emitting fluorophores in the macula of healthy young individuals and to assess the repeatability of these quantitative metrics. Importantly, we explored regional quantitative differences in the distribution of the green-emitting fluorophores.

## 2. Methods

### 2.1. Study Participants

In this observational non-interventional study, healthy volunteers between 18 and 40 years of age were recruited. The San Raffaele Ethics Committee was notified about this observational study, as for Italian legislation. This study adhered to the tenets of the Declaration of Helsinki and Health Insurance Portability and Accountability Act. Written informed consent was obtained from all subjects.

Exclusion criteria were: (i) ocular or medical history of any disorder, including diabetes, diabetic retinopathy, and systemic hypertension; (ii) history of previous ocular surgery. Furthermore, poor-quality images with significant artifacts were excluded. 

All subjects were imaged with the EIDON FAF device (CenterVue, Padua, Italy) between May 2019 and September 2019. For each volunteer, one eye (randomly selected) was imaged. In order to assess the repeatability and to avoid confounding artifacts such as the impact of bleaching or residual activated fluorophores, all subjects were scanned twice but with 7 ± 3 days between scans. Furthermore, all subjects received a complete ophthalmologic examination, which included: (i) measurement of best-corrected visual acuity (BCVA); and (ii) slit lamp anterior segment examination and dilated posterior segment examination.

### 2.2. Spectrally Resolved Fundus Autofluorescence Imaging

Fundus autofluorescence imaging was performed using a confocal light-emitting diode (LED) fundus imaging system (EIDON, CenterVue, Padua, Italy) with a blue illumination wavelength (range 440–475 nm, peak at 450 nm) and emission detection between 500 and 700 nm. Radiant exposure for blue LED lies well below the standard limits established by the American National Standards Institute and other international standards [11,12] Therefore, our protocol may be considered to be safe for clinical imaging.

The examination was performed as previously described [7,8,9,10]. In brief, the device performs autoalignment, autofocus (range −12 to +15 diopters), autoexposure, and autocapture of the images. The autofluorescence emission spectrum is simultaneously measured within a short-wavelength range (500–560 nm, GEFC) and within a long-wavelength range (560–700 nm, REFC) using a line-scanning principle. The resultant FAF image can be displayed as a color-coded image, in which each pixel has a color dependent on the autofluorescence intensity of the two captured spectra/channels. The obtained color-coded FAF image has a frame size of 60 (h) × 55 (w) degrees and a resolution of 4608 × 3288 pixels. The lateral retinal resolution equals 15 microns.

### 2.3. Image Processing

**Figure 1:**
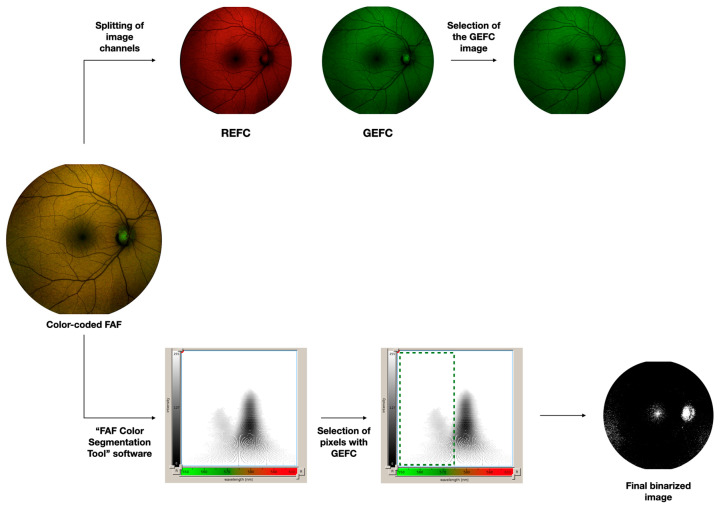
Representation of the algorithm used to process the images.

Color-coded FAF images were then imported in the custom-made analysis software called ‘‘FAF Color Segmentation Tool’’ developed by the instrument manufacturer (CenterVue). As previously reported [9,10]. This software provides an output that displays the fluorescence intensity and emission wavelengths as a two-dimensional graph. Therefore, each pixel of the image is visualized in an xy-graph and for each pixel, measurements of both the autofluorescence intensity and emission wavelengths may be obtained. Furthermore, using this software, those pixels in the color-coded FAF image which are characterized by an autofluorescence falling within a specific intensity and/or wavelength range may be selected and highlighted in a binarized image (Figure 1). Using binarization, all those pixels falling within this selected range are visualized as white, while the other pixels are black. Thus, using this software, we first specifically selected those pixels characterized by a GEFC signal at any intensity, which allowed quantification of the area occupied by green-emitting fluorophores, Quantification was performed by importing these binarized images into ImageJ software version 1.50 (National Institutes of Health, Bethesda, MD, USA; available at http://rsb.info.nih.gov/ij/index.html). 

In order to evaluate the green emission fluorescence component (GEFC) intensity (upper part of the diagram), the color-coded FAF image was exported and then imported into image analysis ImageJ software version 1.50 (National Institutes of Health, Bethesda, MD, USA; available at http://rsb.info.nih.gov/ij/index.html). This image was processed with the “split channels” function, which splits the image into the respective red and green image channels. The GEFC intensity was evaluated as the mean brightness of the green channel image.

The area occupied by green-emitting fluorophores was investigated by importing the color-coded FAF image in the software called “FAF Color Segmentation Tool” and developed by CenterVue. This software grants to visualize fluorescence intensity and emission wavelengths as a two-dimensional graph. We selected those pixels characterized by a GEFC signal and finally obtained a binarized image with these pixels visualized in white.

Second, since the autofluorescence may be characterized by different signal intensities, we also quantified the GEFC intensity (Figure 1). To do so, the color-coded FAF image was also imported into ImageJ. As reported previously [7,8]. The color-coded FAF image was processed with the “split channels” function, which splits the image into the respective red and green image channels (no blue channel is present in this image). In order to assess the GEFC intensity, the mean brightness of the green channel image was calculated as the mean of all the pixel values (considering each pixel in this image may be in a gray scale range of possible values from 0 to 255, where typically 0 is taken to be black, and 255 is taken to be white).

Both the percentage of area occupied by green-emitting fluorophores and the GEFC intensity were assessed in the macular region which was defined as a circle centered on the fovea with a diameter of 6.0 mm. Furthermore, in order to provide a more granular topographical analysis, the analysis of the macular region was further performed in different sub-fields: foveal, parafoveal and perifoveal areas (with diameters of 1.5 mm, 3.0 mm, and 6.0 mm, respectively) [13]. 

### 2.4. Statistical Analysis 

Statistical calculations were performed using Statistical Package for Social Sciences (version 20.0, SPSS Inc., Chicago, IL, USA). 

The level of agreement between repeated measurements was evaluated by assessing the intraclass correlation coefficient (ICC; two-way random, absolute agreement), the 95% coefficient of repeatability (CR), the coefficient of variation (CV), and the mean absolute intraobserver variability.

Related-samples Friedman’s two-way analysis of variance non-parametric test was conducted to compare the measurements among the different macular regions. For the latter evaluation, the color-coded FAF image obtained during the first image session was used.

The Mann–Whitney U Test was used to compare variables between two groups of patients grouped on the basis of age. 

The chosen level of statistical significance was *p* < 0.05.

The sample size of the study was tested to be proper considering a power of 80%.

## 3. Results

Thirty eyes from 30 healthy volunteers (22 males, 8 females) were included in analysis. Mean age was 26.2 ± 2.8 years [median: 25.0 years; range: 23.0–32.0 years]. 

### 3.1. Quantification of the Green-Emitting Fluorophores

**Figure 2:**
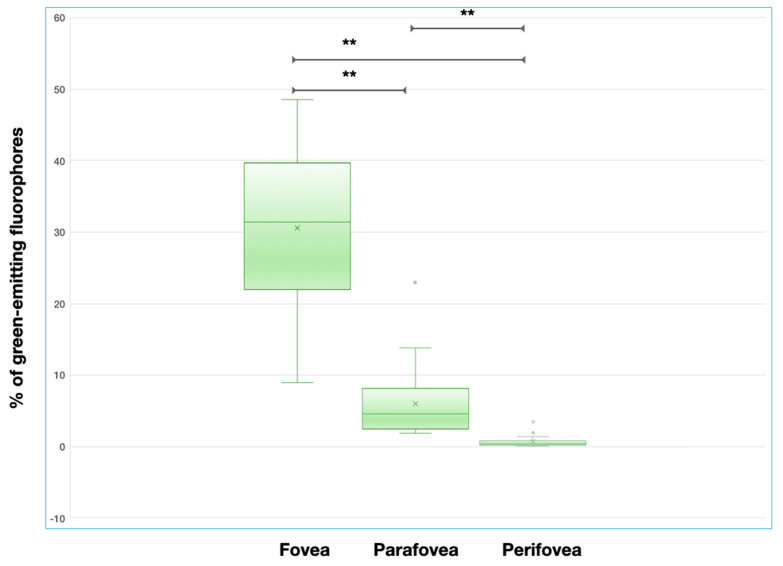
Box and whisker plots showing measurements of the area occupied by green-emitting fluorophores in different analyzed regions. Each box shows median (central horizontal line), mean (“x” in the box) and interquartile range (horizontal extremes of the box) values for each variable. The ends of the whiskers represent the minimum and maximum values. Dots not included in whiskers represent outliers. Horizontal black lines marked with two asterisks indicate statistically significant differences between groups (*p* < 0.0001).

Median (interquartile range, IQR) area occupied by green-emitting fluorophores was 3.6% (1.9–4.7%) in the macular region. In the topographical analysis, this percentage was higher in the foveal region (median = 33.3%, IQR = 21.9–41.2%), as compared with both the parafoveal (median = 5.3%; IQR = 2.4–8.1%; *p* < 0.0001) and perifoveal (median = 0.5%, IQR = 0.2–0.8%; *p* < 0.0001) regions (Figure 2). A significant statistical difference was also found in the comparison between the parafoveal and perifoveal regions (*p* < 0.0001) (Figure 2).

The median intensity of the green emission fluorescence component was 39.4 (IQR = 34.1–45.0) in the foveal region, 60.3 (IQR = 53.8–67.4) in the parafoveal region (*p* < 0.0001 vs. foveal region), and 81.7 (IQR = 74.9–86.2) in the perifoveal region (*p* < 0.0001 vs. foveal region and *p* < 0.0001 vs. parafoveal region).

In a post-hoc analysis, patients were grouped on the basis of age (> or ≤25 years old) yielding two sub-groups. No differences were found between these two groups in terms of area occupied by green-emitting fluorophores in all of the analyzed regions (*p* = 0.325 in the macular area, *p* = 0.215 in the foveal area, *p* = 0.368 in the parafoveal area, and *p* = 0.787 in the perifoveal area). 

### 3.2. Repeatability Analysis

**Figure 3:**
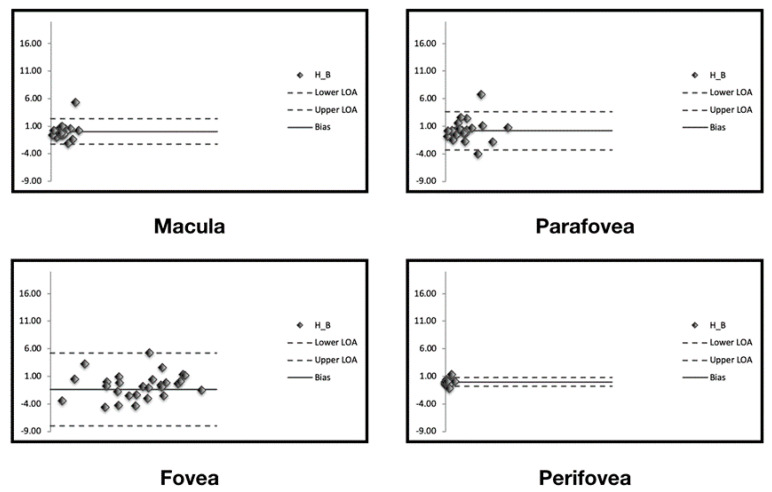
Bland–Altman graphs for repeatability agreement. The Bland–Altman graphs (one for each analyzed region) show the measurement differences for the area occupied by green-emitting fluorophores between two measurements (y-axis) vs. the mean of the two measurements (x-axis). The solid line indicates the mean difference and the dashed lines indicate the 95% limits of agreement.

The level of agreement on assessment of percentage of area occupied by green-emitting fluorophores between repeated measurements is illustrated in the Bland–Altman plots (Figure 3) and in Table 1. 

The mean absolute inter-session variability between measured areas occupied by green-emitting fluorophores was 0.0 for the macular region. The topographical analysis ranged between 0.0 and 1.4 in the topographical analysis (Table 1). 

The CR (the value below which the difference between two measurements will lie in 95% of cases) was 2.3% for the entire macular region. In the topographical analysis, the CR was lowest in the perifoveal region (Table 1). 

Likewise, the CV (ranging from 1.1 to 1.7%) and ICC (ranging from 0.93 to 0.97) indicated that the quantification of green-emitting fluorophores is characterized by high levels of repeatability. 

In the GEFC intensity assessment, the mean absolute inter-session variability was 5.3, 7.4 and 5.7 for the foveal, parafoveal, and perifoveal regions, respectively. The CR was 2.3 in the fovea, 2.1 in the parafovea, and 0.9 in the perifovea. Likewise, the CV (0.2 in the foveal region, 0.1 in the parafoveal region, and 0.1 in the perifoveal region) and ICC (ranging from 0.91 to 0.96) displayed high levels of repeatability in this assessment. 

## 4. Discussion

In this study, we report quantitative data and repeatability of green-emitting fluorophores in healthy eyes using a shorter wavelength (~450 nm) spectrally resolved LED blue-light confocal FAF system. Overall, we observed that the distribution of these fluorophores is highest in the foveal region. Importantly, we demonstrated that these measurements have high levels of repeatability. 

This technology has recently been used to investigate diseased eyes [7,8,9,10]. A recent report of eyes with atrophy from age-related macular degeneration (AMD) demonstrated that in areas of atrophy the strong red emission fluorescence component from lipofuscin was absent or reduced, while residual green-emitting fluorophores were detected corresponding to subretinal hyperreflective material and believed to represent drusenoid material partially composed of AGE [7]. Successively, it was demonstrated that the increased excitation of green-emitting fluorophores in the region of AMD-associated atrophy may negatively impact the capability of this shorter wavelength FAF for detection and measurement of atrophy [8]. However, the isolation of the REFC from the “color-coded” FAF images granted the identification of atrophic lesions and an accurate and reproducible quantification of regions of atrophy [8]. The assessment of ABCA4-related retinopathy with this technology revealed that flecks are heterogeneous in terms of fluorescence emission [9]. Specifically, the centrally located flecks were characterized by a higher autofluorescence emission of green-emitting fluorophores (or GEFC), and these flecks were morphologically characterized by subretinal deposits disrupting the outer retinal layer integrity. In contrast, peripheral flecks were observed to have predominantly red-emission fluorescence and showed a lower disturbance of the outer retinal layers on structural OCT. Finally, a study employing this spectrally-resolved FAF technology in eyes with optic disc drusen revealed that these lesions are distinguished by an increased autofluorescence in the green-emission component [10].

As noted above, the main green-emitting fluorophores include FAD, AGE, and collagen/elastin [4]. FAD is an intracellular molecule stored in mitochondria and, therefore, has been suggested as a potential biomarker for metabolic and mitochondrial activities [14]. AGE and collagen/elastin are extracellular proteins. Several studies have demonstrated that AGE are a group of proteins which play a role in aging changes and are also involved in AMD and diabetic retinopathy pathogenesis [13,15,16]. The posterior eye is also rich in collagen- and elastin-rich extracellular matrix, which is mainly contained in Bruch’s membrane, choroid and sclera [17,18]. 

Based on our study, we are now able to report on the macular distribution of green-emitting fluorophores in a healthy population. Analysis of the distribution demonstrated that these fluorophores are more prominent in the foveal region, in comparison with both the parafoveal and perifoveal areas. As noted above, FAD is one of the main green-emitting fluorophores and may be considered as a surrogate for the presence of mitochondria in the retinal cells. Furthermore, the inner segments of the photoreceptor cells are known to be rich in mitochondria which is essential to provide the energy required by the ionic pumps that drive the ‘dark current’ [19]. Notably, cones were demonstrated to have an increased density of mitochondria as compared with rods, because of their enhanced metabolic demand, as well as to ameliorate their waveguide properties [19]. Based on these observations, and given that we employed a confocal device which collected data from the focal plane focused at the outer retina and RPE level, one potential hypothesis is that our finding of an increased distribution of green-emitting fluorophores in the foveal region may be related to the higher density of cones in the same area. 

Of note, the green-emission fluorescence component may be detected with variable intensity. Our data demonstrated that, although the fluorescence distribution of the green-emitting fluorophores was highest in the foveal region, their fluorescence intensity was reduced in this area. Macular pigment is known to attenuate the ocular autofluorescence by absorbing the short-wavelength excitation light and, as a result, this causes a reduction of the green emission fluorescence component intensity in this central region [20]. 

In the present study, we also assessed the repeatability of macular green-emitting fluorophores’ measurements. Repeatability refers to the agreement of measurements from different sessions of scans using the same device, same eye, and same operator within a short period. Notably, we demonstrated that the quantification of green-emitting fluorophores has a high level of repeatability in all the analyzed regions. In detail, we found an intraclass correlation coefficient greater that 0.90 and a CR <6.6% in all analyzed regions, which suggests an excellent repeatability of these measurements [21].

Our study has limitations which should be considered when interpreting our results. First, the sample size of the cohort is relatively small which reduces the power of our analysis. Second, although we have speculated that the topographical distribution of the green-emitting fluorophores may reflect the heterogeneous distribution of mitochondria in photoreceptors, other fluorophores beyond FAD could also contribute to the GEFC. Even though AGE is mainly present in diseased eyes and collagen should be equally distributed in the posterior pole, we are not able to exclude their contribution to the GEFC signal. Moreover, our study was aimed at quantifying green-emitting fluorophores in healthy young subjects (mean ± SD age of 26.2 ± 2.8 years) and we thus included subjects with a narrow range of age. Thus, although we performed a post-hoc analysis grouping patients on the basis of age, we are not able to define the impact of age on the quantity and distribution of green-emitting fluorophores based on the present study.

In summary, using a novel shorter wavelength (~450 nm) spectrally resolved FAF system, the distribution of green-emitting fluorophores in the eye can be probed in more detail. The distribution of these “minor” fluorophores in healthy eyes appear to have a topographical variability. We speculate that this variability could be secondary to an increased quantity of mitochondria in the foveal region which features a high cone density. The potential role of quantitative green-emission fluorescence for assessment of retinal metabolic status warrants further study.

## Figures and Tables

**Table 1:** Bland–Altman graphs for repeatability agreement.

	Macula	Fovea	Parafovea	Perifovea
Mean absolute intraobserver variability	0.0	1.4	0.2	0.0
CR	2.3	6.6	3.5	0.8
CV	1.7	1.1	1.4	1.6
ICC	0.93	0.97	0.96	0.96

CR: 95% coefficient of repeatability; CV: coefficient of variation; ICC: intraclass correlation coefficient; GA: geographic atrophy; IQR: interquartile range.
